# Elevated Expression of Cathepsin K in Periodontal Ligament Fibroblast by Inflammatory Cytokines Accelerates Osteoclastogenesis via Paracrine Mechanism in Periodontal Disease

**DOI:** 10.3390/ijms22020695

**Published:** 2021-01-12

**Authors:** Soon Chul Heo, Yu Na Kim, YunJeong Choi, Ji-Young Joo, Jae Joon Hwang, Moon-Kyoung Bae, Hyung Joon Kim

**Affiliations:** 1 Department of Oral Physiology, Periodontal Diseases Signaling Network Research Center, Dental and Life Science Institute, School of Dentistry, Pusan National University, Yangsan 50612, Korea; snchlheo@gmail.com (S.C.H.); kyn0394@naver.com (Y.N.K.); celinechoi@pusan.ac.kr (Y.C.); mkbae@pusan.ac.kr (M.-K.B.); 2 Department of Periodontology and Dental Research Institute, Pusan National University Dental Hospital, Yangsan 50612, Korea; joojy@pusan.ac.kr; 3 Department of Oral and Maxillofacial Radiology and Dental Research Institute, Pusan National University, Yangsan 50612, Korea; softdent@pusan.ac.kr

**Keywords:** cathepsin K, periodontal disease, periodontal ligament fibroblast, inflammatory cytokine, osteoclastogenesis

## Abstract

Cathepsin K (CTSK) is a cysteine protease that is mainly produced from mature osteoclasts and contributes to the destruction of connective tissues and mineralized matrix as a consequence of periodontal disease (PD). However, few studies have reported its regulatory role in osteoclastogenesis-supporting cells in inflammatory conditions. Here, we investigated the role of CTSK in osteoclastogenesis-supporting cells, focusing on the modulation of paracrine function. Microarray data showed that CTSK was upregulated in PD patients compared with healthy individuals, which was further supported by immunohistochemistry and qPCR analyses performed with human gingival tissues. The expression of CTSK in the osteoclastogenesis-supporting cells, including dental pulp stem cells, gingival fibroblasts, and periodontal ligament fibroblasts (PDLFs) was significantly elevated by treatment with inflammatory cytokines such as TNFα and IL-1β. Moreover, TNFα stimulation potentiated the PDLF-mediated osteoclastogenesis of bone marrow-derived macrophages. Interestingly, small interfering RNA-mediated silencing of CTSK in PDLF noticeably attenuated the TNFα-triggered upregulation of receptor activator of nuclear factor kappa-B ligand (RANKL), macrophage colony-stimulating factor, and RANKL/osteoprotegerin ratio, thereby abrogating the enhanced osteoclastogenesis-supporting activity of PDLF. Collectively, these results suggest a novel role of CTSK in the paracrine function of osteoclastogenesis-supporting cells in periodontal disease.

## 1. Introduction

Periodontal diseases (PDs) are infectious and chronic inflammatory disorders that destroy the tooth-supporting tissues [1]. The neglect of oral hygiene may cause an oral microbiome imbalance, which can be identified by detecting immunoglobulin antibodies, leading to a deterioration in oral health-related quality of life [2]. Dental plaque, which is a biofilm composed of pathogenic periodontal bacteria, has been suggested to initiate PDs by releasing several detrimental compounds, such as free radicals, collagen-destroying enzymes, and toxins [3]. The bacteria and their products from dental plaque stimulate inflammatory reactions during PD progression by activating the host immune response. Several kinds of pro-inflammatory cytokines have been reported to be involved in this process, such as IL-6, IL-1, and TNF families [4,5,6]. The breakdown of the delicate balance between the local immune response and the microbiota in homeostasis of periodontal tissue leads to an increase in osteoclast activity, which can destroy periodontal connective tissue and alveolar bone [7].

Osteoclasts are multinucleated cells characterized by their ability to resorb bone or mineralized dentin matrix. They are differentiated either from hematopoietic monocyte/macrophage lineage precursors or from tissue-resident embryo-derived precursors through osteoclastogenesis [8,9]. Under inflammatory conditions, pro-inflammatory cytokines stimulate the secretion of receptor activator of nuclear factor kappa-B ligand (RANKL) and macrophage colony-stimulating factor (M-CSF), which are the central regulators of osteoclastogenesis from osteoblasts and stromal cells, while osteoprotegerin (OPG), a soluble decoy receptor, abrogates osteoclast differentiation by competing with RANK for RANKL [10,11]. RANKL binds to RANK on the extracellular surface of osteoclast precursors and activates NFATc1 by stimulating c-Fos [12], which induces bone resorption and cell fusion activity by upregulating the expression of osteoclast marker genes, such as tartrate-resistant acid phosphatase (TRAP), cathepsin K (CTSK), and dendritic cell-specific transmembrane protein (DC-STAMP) [13,14].

Many studies have demonstrated that several kinds of proteases are involved in bone resorption. Among them, CTSK is predominantly expressed in osteoclasts and positively associated with bone resorption [15]. CTSK, a lysosomal cysteine protease belonging to the peptidase C1 protein family, has become one of the most attractive therapeutic targets due to its remarkable bone resorptive activity [16]. Accumulating evidence suggests that CTSK is involved in various diseases beyond bone-resorptive disorders such as stroke [17], atherosclerosis [18], fibrosis [19], and arthritis [20]. During physiological root resorption, CTSK, expressed in the odontoclasts mediates the extracellular degradation of dentin collagen [21]. In addition, the expression of CTSK in gingival crevicular fluid is positively correlated with the severity of periodontitis, suggesting a stimulatory effect of CTSK on the progression of PDs [22]. However, the regulatory role of CTSK in modulating paracrine function of osteoclastogenesis-supporting cells and its source during PD development have not yet been fully evaluated.

In this study, we observed the non-osteoclastic upregulation of CTSK in periodontitis patients. TNFα and IL-1, which are key pro-inflammatory cytokines produced during PD progression, upregulated the expression of CTSK in dental pulp stem cells (DPSCs), gingival fibroblasts (GFs), and periodontal ligament fibroblasts (PDLFs). The results from the transwell co-culture system demonstrated that TNFα-activated PDLF potentiated the RANKL-induced osteoclastogenesis in mouse bone marrow-derived macrophages (BMMs). The silencing of CTSK with CTSK-specific small interfering RNA (siRNA) in PDLF not only abrogated the TNFα-induced expression of M-CSF and RANKL but also attenuated the paracrine effects of PDLF on osteoclastogenesis in BMMs. Taken together, our results suggest a novel modulatory role of CTSK on the paracrine function of osteoclastogenesis-supporting cells.

## 2. Results

### 2.1. Gingival Tissue from PD Patients Has Increased Levels of CTSK

**Figure 1:**
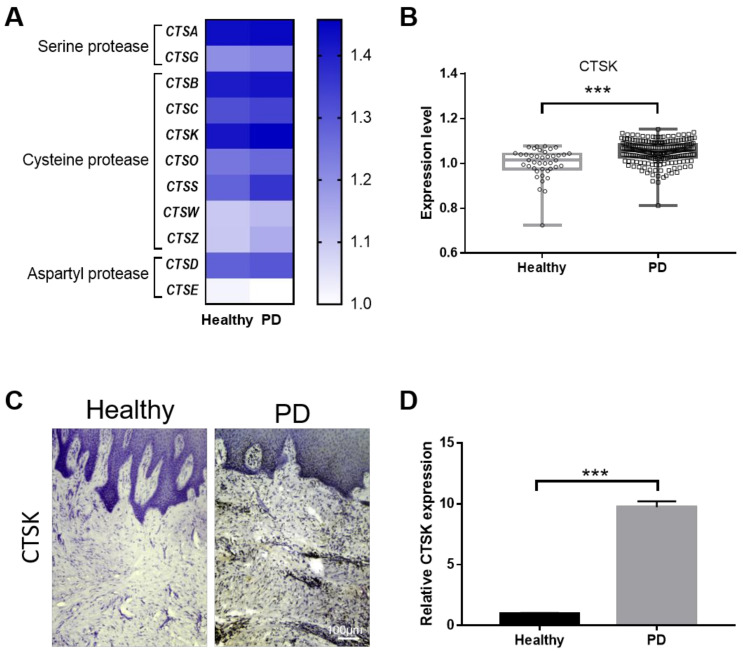
Comparison of cathepsin expression in healthy individuals and patients with periodontal disease. (**A**) The expression profiles of the members of the cathepsin family in E-MTAB. (**B**) Comparison of CTSK expression in healthy donors (*n* = 76) and patients with periodontal disease (*n* = 224). Immunohistochemical analysis (**C**) and mRNA expression (**D**) of CTSK in healthy donors and patients with PD. Data represent the mean ± S.E.M. *** *p* < 0.001 by Student’s *t*-test.

To examine whether cathepsin isoforms are elevated in periodontal disease, we first analyzed a microarray dataset from a public database. Eleven cathepsin isoforms were identified from the dataset, and 10 out of 11 genes were upregulated in PD patients (Figure 1A). Among them, we focused on the expression of CTSK since CTSK is well known for its bone resorptive activity in PD. The microarray dataset showed that the expression of CTSK was significantly higher in individuals with PD than in healthy individuals (Figure 1B). To further confirm the microarray data, we performed immunohistochemistry and quantitative real-time polymerase chain reaction (qPCR) with gingival tissue samples obtained from healthy individuals and PD patients. Immunohistochemical staining showed a strong expression of CTSK in the gingiva from PD patients throughout the periodontal tissues, compared to that of normal gingiva (Figure 1C). qPCR analysis further confirmed that the expression of CTSK was dramatically increased in gingival tissues from patients with PD than in normal samples (Figure 1D). These results strongly suggest that CTSK expression is increased in the gingiva of PD patients.

### 2.2. Administration of TNFα and IL-1β Elevates the Expression of CTSK in Osteoclastogenesis-Supporting Cells

**Figure 2:**
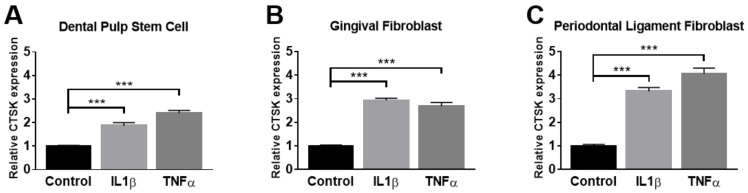
The expression of CTSK in response to inflammatory cytokine in osteoclastogenesis-supporting cells. Three types of osteoclastogenesis-supporting cells, including dental pulp stem cells (**A**), gingival fibroblasts (**B**), and periodontal ligament fibroblasts (**C**), were treated with 10 ng/mL of TNFα or IL-1β for 24 h. The mRNA expression of CTSK was determined by qPCR and normalized to the expression levels of β-actin. Data represent the mean ± S.E.M. *** *p* < 0.001 by one-way ANOVA.

TNFα and IL-1β have been well characterized as key cytokines in inflammatory reactions in the PD microenvironment. To examine whether TNFα and IL-1β could enhance the expression of CTSK in osteoclastogenesis-supporting cells in vitro, the expression of CTSK upon treatment with TNFα or IL-1β was analyzed in several osteoclastogenesis-supporting cells localized in the periodontium, including DPSCs, GFs, and PDLFs. The results revealed a prominent increase in CTSK expression upon TNFα and IL-1β stimulation in all examined cells (Figure 2A–C). Among them, the responsiveness of PDLF against TNFα was the most potent. Thus, we used PDLF and TNFα in subsequent experiments. These results suggest that pro-inflammatory cytokines enhance the expression of CTSK in osteoclastogenesis-supporting cells in vitro, resembling the pathological response that emerges in the PD microenvironment in vivo.

### 2.3. TNFα Enhances the Osteoclastogenesis-Supporting Activity of PDLFs

**Figure 3:**
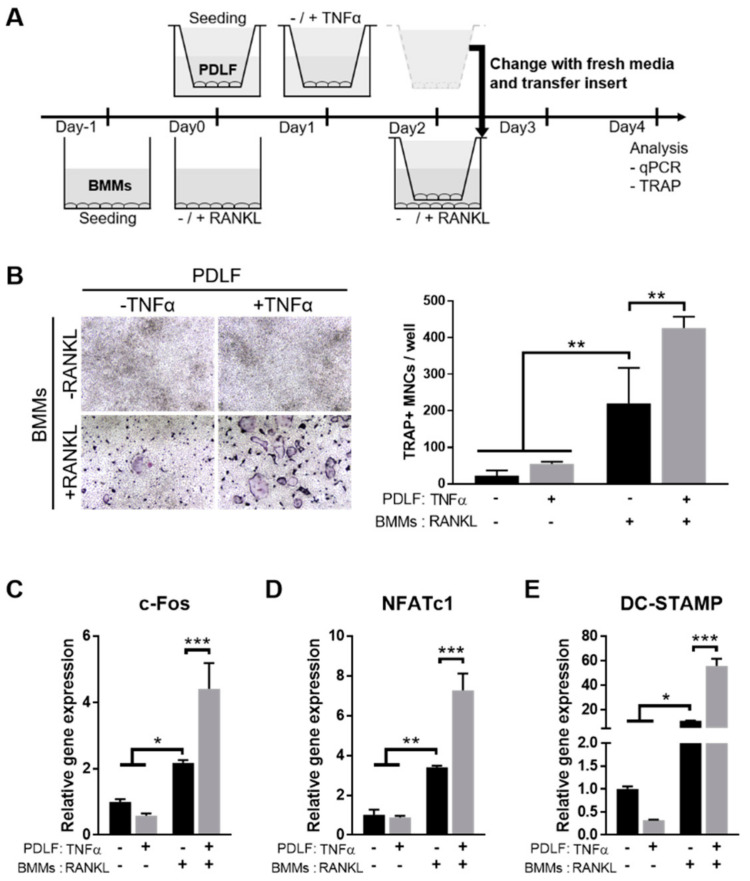
The TNFα-stimulated PDLF showed increased osteoclastogenesis-supporting activity (**A**) A schematic diagram of the experimental procedure. PDLF (upper chamber) was activated with TNFα for 24 h prior to co-culture with BMMs (lower chamber). In this co-culture system, osteoclast differentiation was allowed in the presence or absence of 100 ng/mL of RANKL as indicated. (**B**) Representative images of TRAP staining (left) and quantitative analysis (right) of TRAP-positive MNCs. (**C**–**E**) The expression of osteoclastogenic markers including c-FOS, NFATc1, and DC-STAMP in BMMs was determined by qPCR and normalized to the expression levels of β-actin. Data represent the mean ± S.E.M. * *p* < 0.05, ** *p* < 0.01, and *** *p* < 0.001 by two-way ANOVA. MNCs, mononuclear cells.

Next, to examine the effects of TNFα-activated PDLF on osteoclastogenesis, which is one of the key pathological processes of PD development, we adopted the transwell co-culture system using mouse BMMs and PDLF. The BMMs were seeded on the lower chamber, treated with EANKL for 48 h to allow differentiation into osteoclasts and then co-cultured with TNFα-activated PDLF seeded on the upper chamber (Figure 3A). The TRAP staining results showed that the TNFα-activated PDLF further increased the number of TRAP-positive multi-nucleated cells (MNCs), which was more conspicuous in the presence of RANKL (Figure 3B). Consistent with the TRAP staining results, the osteoclast marker genes, including c-FOS, NFATc1, and DC-STAMP, were significantly upregulated in the co-culture with TNFα-activated PDLFs compared to non-activated-PDLFs (Figure 3C–E). These results indicate that TNFα-activated PDLFs could potentiate RANKL-induced osteoclastogenesis of BMMs through a paracrine mechanism.

### 2.4. TNFα-Stimulated Expression of Osteoclastogenesis-Modulating Factors Were Ameliorated by CTSK-Silencing in PDLFs

**Figure 4:**
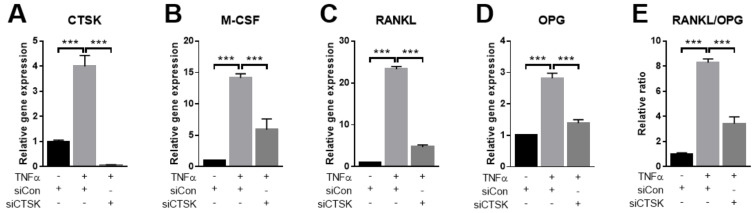
Effects of CTSK silencing on the expression of osteoclastogenesis-modulating factors. PDLFs were transfected with either siControl or siCTSK and then treated with 10 ng/mL of TNFα for 24 h. The mRNA expression levels of CTSK (**A**), M-CSF (**B**), RANKL (**C**), and OPG (**D**) were determined by qPCR and normalized to the expression levels of β-actin. (**E**) Quantitative analysis of the RANKL/OPG ratio. Data represent the mean ± S.E.M. *** *p* < 0.001 by one-way ANOVA.

As TNFα stimulated the expression of CTSK and activated the paracrine osteoclastogenesis-supporting activity of PDLFs in previous studies, we hypothesized that CTSK could modulate the paracrine function of PDLFs. M-CSF, RANKL, and OPG are crucial osteoclastogenesis modulating factors that are released from osteoclastogenesis-supporting cells and contribute to osteoclastogenesis during PD development [23]. To examine this hypothesis, we examined the change in osteoclastogenesis modulating gene expression following depletion of CTSK by siRNA in PDLFs. The TNFα-induced mRNA expression of CTSK was completely abrogated by CTSK-specific siRNA, as confirmed by qPCR (Figure 4A). Interestingly, transfection with CTSK-specific siRNA resulted in marked attenuation of TNFα-induced mRNA expression of M-CSF, RANKL, and OPG (Figure 3B–D). In addition, the RANKL/OPG ratio, which is an important determinant of osteoclast differentiation [24], was also decreased by CTSK silencing. These results suggest a novel role of CTSK in modulating osteoclastogenesis in osteoclastogenesis-supporting cells activated by inflammatory cytokines.

### 2.5. CTSK Is Responsible for TNFα-Induced Paracrine Function of PDLFs in Osteoclastogenesis

**Figure 5:**
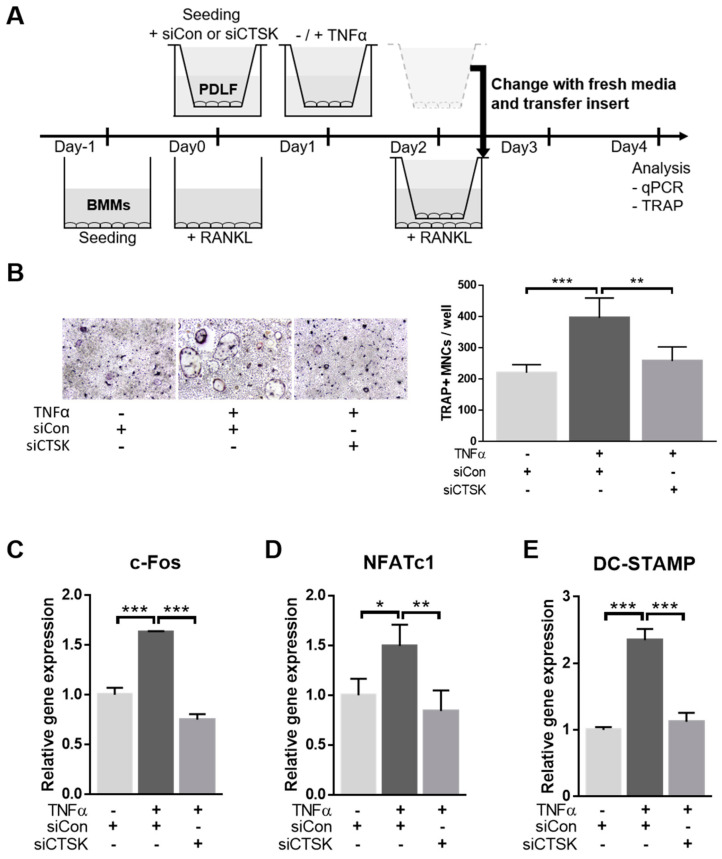
The involvement of CTSK on TNFα-induced stimulatory effect of PDLFs in osteoclastogenesis. (**A**) A schematic diagram of the experimental procedure. PDLFs transfected with either siControl or siCTSK were seeded on the upper chamber of the transwell. After 24-h treatment with TNFα, the PDLF-seeded upper chamber was transferred onto the BMM-seeded lower chamber. Osteoclastogenesis was induced with 100 ng/mL of RANKL every other day for a 4-day period. (**B**) Representative images of TRAP staining (left) and quantitative analysis (right) of TRAP-positive MNCs. (**C**–**E**) The mRNA expression of osteoclastogenic markers, including c-FOS, NFATc1, and DC-STAMP in BMMs, was determined by qPCR and normalized to β-actin expression. Data represent the mean ± S.E.M. * *p* < 0.05, ** *p* < 0.01, and *** *p* < 0.001 by one-way ANOVA.

To explore whether CTSK is responsible for the stimulatory effect of TNFα-activated PDLFs on osteoclastogenesis, BMMs were co-cultured with CTSK-silenced PDLFs in a transwell system. PDLFs were transfected with siControl or siCTSK separately (upper chamber) and then transferred onto RANKL-treated BMMs (lower chamber) (Figure 5A). Consistent with previous observations, TNFα-activated PDLFs significantly increased the number of TRAP-positive MNCs under osteoclastogenesis induction. Silencing of CTSK, however, resulted in markedly decreased TNFα-activated PDLF-induced osteoclastogenesis (Figure 5B). The qPCR analysis results indicated that the paracrine stimulation of c-FOS, NFATc1, and DC-STAMP expression by TNFα-activated PDLFs was also dramatically decreased by CTSK silencing. These results suggest a crucial role of CTSK in the elevation of osteoclastogenesis-supporting activity of PDLFs upon TNFα stimulation, which is mainly mediated by the paracrine action of PDLF-secreted osteoclastogenesis-modulating factors.

### 2.6. CTSK Is Involved in TNFα-Activated Paracrine Function of PDLF in Bone Resorption

**Figure 6:**
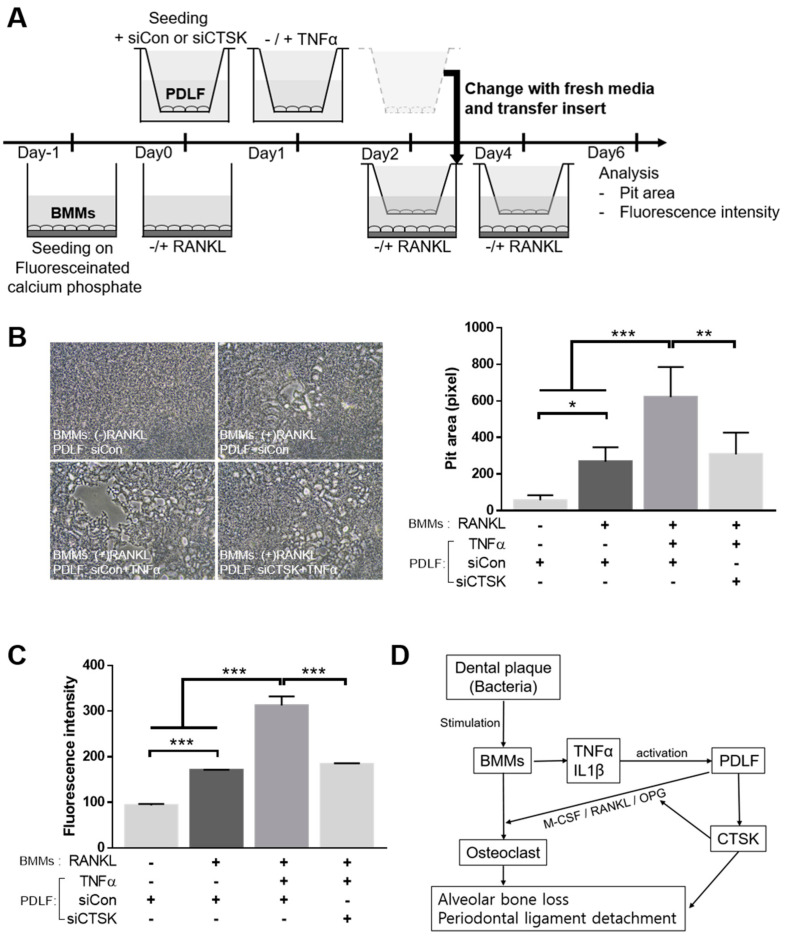
The role of CTSK in the TNFα-stimulated paracrine regulation of bone resorption. (**A**) A schematic diagram of the experimental procedure. PDLFs transfected with either siControl or siCTSK were seeded on the upper chamber of the transwell. Upon 24-h treatment with TNFα, the PDLF-seeded upper chamber was transferred to the BMM-seeded lower chamber coated with fluorescein-labeled calcium phosphate. Osteoclastogenesis was induced with 100 ng/mL of RANKL every other day and analyzed on day 6. (**B**) Representative microscopic images (left) and quantitative analysis (right) of the pit area. (**C**) The quantitative analysis of the fluorescence intensity in the conditioned medium. (**D**) A schematic diagram showing the novel suggested regulatory mechanism of CTSK in the PD microenvironment. CTSK expression is stimulated by inflammatory cytokines in osteoclastogenesis-supporting cells. CTSK could contribute to the deterioration of PD either directly via involvement of alveolar bone loss and periodontal ligament detachment or indirectly via modulation of the osteoclastogenesis-regulating factor. Data represent the mean ± S.E.M. * *p* < 0.05, ** *p* < 0.01, and *** *p* < 0.001 by one-way ANOVA.

To examine how CTSK modulates the TNFα-induced paracrine function of PDLF and affects the resorption activity of osteoclasts, a bone resorption assay was performed, as shown in Figure 6A. Consistent with the TRAP staining results, TNFα-further increased the PDLF-stimulated pit area formation, and this increase was significantly attenuated by CTSK silencing (Figure 6B). Moreover, the results of fluorescent intensity, a quantitative reflection of bone resorption activity using released calcium phosphate in the conditioned medium, also showed that the silencing of CTSK dramatically abrogated the stimulatory effect of TNFα-activated PDLF on fluorescence intensity promotion. These results suggest that CTSK contributes to the stimulation of resorbing activity by modulating the paracrine function of osteoclastogenesis-supporting cells under inflammatory conditions (Figure 6D).

## 3. Discussion

In the present study, we showed novel stimulatory effects of CTSK on the paracrine function of osteoclastogenesis-supporting cells. During PD progression, CTSK was previously believed to be expressed mainly in mature osteoclasts and contribute to the devastation of periodontal tissue via detachment of the periodontal ligament or alveolar bone loss. Although various sources of non-osteoclastic CTSK have been reported in pathologic environments, such as the synovium of patients with rheumatoid arthritis (RA) [25], smooth muscle cells in atheroma [26], and articular hyaline cartilage in osteoarthritis [27], the non-osteoclastic cellular sources of CTSK in PD have not been fully explored. Several investigators have suggested that gingival crevicular fluid (GCF) is a useful predictor in determining the severity of PD [28,29]. The increased concentration of CTSK in GCF was positively correlated with periodontitis. Beklen et al. have also demonstrated that GCF-CTSK is derived from fibroblast-like cells, vascular endothelial cells, and gingival epithelial cells in periodontitis and contributes to the loss of attachment and destruction of the periodontal ligament [30]. The GCF localizes in the gingival crevice, which provides a slightly acidic pH optimal for CTSK to actively degrade the extracellular matrix [31]. In addition, the gingival crevice represents an ideal environment for the oral microbiota that forms a dental biofilm on the tooth surface due to its rich source of nutrients and anaerobic conditions [32]. In the current study, we provided evidence that the expression of CTSK in osteoclastogenesis-supporting cells, including DPSCs, GFs, and PDLFs, could be activated by TNFα and IL-1β, which are present at high levels in diseased periodontal tissues. These findings suggest that activated osteoclastogenesis-supporting cells, via inflammatory stimuli, could release the CTSK into the extracellular milieu such as gingival crevice, thereby contributing to the acceleration of PD progression by promoting extracellular matrix degradation.

More than a century ago, William Hunter first proposed the concept that linked oral sepsis with diseases of other organs, termed the theory of “focal infection” or “focal sepsis” [33]. However, this theory fell into disrepute due to a lack of scientific rigor. Clear evidence with well-designed studies suggesting the possible linkages between PD and systemic diseases began to emerge from the late 1980s [34]. Since then, a number of studies have further supported the potential association of PDs with other systemic diseases, including atherosclerotic cardiovascular disease [35,36], diabetes [37], and RA [38]. As RA and PD share many common pathological features, such as increased cytokine production, inflammatory cell infiltration, and osteoclastic activity, the associations between these two diseases have been particularly well characterized. CTSK has been described as a critical mediator in the dysregulation of the Toll-like receptor (TLR) pathway, which has been proposed as an inflammatory driver in both RA and PD [39,40]. The deficiency of CTSK led to a drastic decrease in cytokine expression, bone erosion, and osteoclast numbers via the TLR signaling pathway. Consistent with this view, our present findings suggest that CTSK, produced by inflammatory cytokines in osteoclastogenesis-supporting cells during PD, may act as a key factor in the association of PD and systemic diseases.

As CTSK bears the most potent protease activity in mediating bone resorption, it has become the most attractive target for anti-osteoporosis drug development. Although many pharmaceutical companies have attempted to develop selective inhibitors for CTSK for clinical use, no drug has been approved due to the occurrence of undesirable adverse effects [16]. In parallel with siRNA-mediated CTSK silencing, we attempted to inhibit CTSK expression with CTSK-specific inhibitors, Odanacatib and L006235, as well as E-64 (an irreversible cysteine protease inhibitor), to examine the therapeutic potential of CTSK inhibitors. Interestingly, all the tested inhibitors further increased the mature form of CTSK protein upon TNFα treatment in PDLF, rather than inhibition (data not shown). This interesting observation indicates that the inhibition of CTSK may trigger a compensatory mechanism, which needs to be clarified further.

Several studies have suggested a possible paracrine effect of PDLFs in osteoclastogenesis via RANKL and OPG regulation [24,41]. It has been shown that the expression of RANKL or OPG in PDLF could be modulated by the virulence factors of periodontal pathogens and inflammatory cytokines. Consistent with previous reports, our study demonstrated that TNF-α stimulation significantly increased the mRNA expression of RANKL, as well as the RANKL/OPG ratio, in PDLFs and further promoted osteoclastogenesis in BMMs via a paracrine mode of action. Interestingly, the TNFα-induced upregulation of RANKL and OPG expression was dramatically depleted upon siRNA-mediated silencing of CTSK. To the best of our knowledge, no other study has shown that CTSK modulates RANKL and OPG at the transcriptional level. A possible mechanism is thought to be the CTSK/TGFβ1/RANKL signaling axis. PDLFs are a dynamic cell population that constantly reorganizes the cytoskeleton to allow the teeth to withstand the physical forces generated during mastication [42]. TGFβ1 is known to be a key molecule responsible for cytoskeletal reorganization [43] and is abundantly expressed in PDLF [44]. Moreover, TGFβ1 showed dose-dependent inhibitory effects on osteoclastogenesis via suppression of the RANKL/OPG ratio in stromal cells [45]. Moreover, TGFβ1 was inversely correlated with CTSK expression during silica-induced lung fibrosis in mice [46]. Collectively, the increased expression of CTSK in response to TNFα may negatively regulate TGFβ1 expression, thereby promoting the expression of RANKL via elimination of the suppressor. Further studies should be conducted to verify the exact mechanism underlying CTSK-dependent modulation of osteoclastogenesis-regulating factors.

The present study demonstrates a novel role of CTSK in modulating the paracrine function of osteoclastogenesis-supporting cells. Depletion of CTSK attenuated the TNFα- and IL-1β-induced M-CSF, RANKL, and OPG expression in PDLF. Moreover, the stimulatory effects of TNFα on PDLF-mediated osteoclastogenesis of BMMs were dramatically reduced by CTSK silencing. Beyond the conventional concepts of CTSK function, these results provide novel insights into the role of CTSK in modulating the paracrine function of osteoclastogenesis-supporting cells during periodontal disease development.

## 4. Materials and Methods

### 4.1. Reagents

Minimum Essential Medium with alpha modifications (α-MEM, #LM008-01) and Dulbecco’s phosphate-buffered saline (DPBS, #LB001-01) were purchased from WelGENE Inc. (Daegu, Korea). Fetal bovine serum (FBS, #16000044), trypsin-EDTA (#25200056), and penicillin-streptomycin (#15140122) were purchased from Gibco (Thermo Fisher Scientific, Inc., Waltham, MA, USA). Recombinant human TNFα (#300-01A), IL-1β (#200-01B), and M-CSF (#300–25) were purchased from Peprotech (Rocky Hill, NJ, USA). Recombinant mouse RANKL (#cyt-320) was purchased from Prospec (Israel). Anti-cathepsin K (#ab19027) antibody was purchased from Abcam (Cambridge, MA, USA). TRAP staining kit (#387A) was purchased from Sigma (St. Louis, MO, USA). Culture plates were purchased from Nunc (Roskilde, Denmark), and Transwell (#37024) was purchased from SPL (Pocheon, Korea).

### 4.2. Cell Culture and Cytokine Treatment

Human dental pulp stem cells were purchased from Lonza (#PT-5025), and human periodontal ligament fibroblasts (#2630) and human gingival fibroblasts (#2620) were purchased from Sciencell (Carlsbad, CA, USA). All cells were cultured in α-MEM supplemented with 10% FBS and 100 U/mL of penicillin-streptomycin at 37 °C in an atmosphere containing 5% CO_2_. Cells used in all experiments were below seven passages. The cells were treated with 10 ng/mL of TNFα and IL-1β for 24 h under serum-free conditions.

### 4.3. BMM Isolation and Osteoclastogenic Cultures

BMMs were purified and used as precursor cells for osteoclasts, as previously described [47]. Briefly, the femora and tibiae were dissected from 6-week-old ICR mice. The animal experiments were approved by the Committee on the Care and Use of Animals in Research, Pusan National University (PNU-2020-2576). Whole bone marrow cells were flushed out with 1 mL of α-MEM and cultured on 100-mm culture dishes for 1 day. The next day, attached stromal cells were discarded, and floating monocytes were harvested and re-plated on Petri dishes in the presence of M-CSF (30 ng/mL) for 3 days. On day 3, the adherent macrophages were detached by mechanical scraping and reseeded to induce osteoclast differentiation. BMMs were allowed to differentiate into osteoclasts in osteoclastogenic medium (30 ng/mL of M-CSF and 100 ng/mL of RANKL) for 4 days, and the medium was changed every other day. At the end of the culture period, osteoclastogenic differentiation was assessed by TRAP staining, and TRAP-positive giant cells with ≥3 nuclei were regarded as osteoclasts.

### 4.4. Public Data Acquisition and Processing

The microarray dataset of E-MTAB-3267 [48] was obtained from the ArrayExpress database. Samples from 76 healthy individuals and 224 individuals with periodontal disease were analyzed.

### 4.5. Immunohistochemistry

Human gingival tissue samples were obtained from the premolars of patients (three clinically healthy gingivae and three periodontitis) during routine periodontal flap surgery at Pusan National University Dental Hospital (PNUDH; IRB number: PNUDH-2020-002). Periodontitis was diagnosed by a pocket depth of ≥4 mm, attachment loss of ≥3 mm, and bleeding upon probing. Human gingival tissues were fixed with 4% PFA, embedded in OCT compound, and then sliced to obtain 10-µm sections using a microtome. For immunohistochemical analysis, sections of OCT-embedded tissues were incubated with CTSK antibodies, followed by incubation with HRP-conjugated secondary antibodies (Vector, #MP-7401). Sections were incubated with the substrate DAB (Vector, #SK-4100) and counterstained with Mayer’s hematoxylin. Digital images of the histochemically stained sections were obtained using a microscope (Nikon, Eclipse Ts2).

### 4.6. qPCR

Total RNA was purified using the RNeasy Mini Kit (Qiagen, #74106) according to the manufacturer’s instructions, and 2 μg of RNA was reverse-transcribed under standard conditions with Superscript II (Invitrogen). For quantitative real-time PCR analysis, 50 ng of cDNA was mixed with SYBR Green PCR Master Mix (Applied Biosystems, Forster, CA, USA) and amplified for 40 cycles using the AB7500 (Applied Biosystems). Experiments were performed in triplicate, and the data were normalized to the expression of β-actin mRNA. Data were analyzed using the Δ(ΔCT) method. The primer sequences used in real-time PCR were as follows: mouse c-FOS: 5′-ACTTCTTGTTTCCGGC-3′, 5′-AGCTTCAGGGTAGGTG-3′; mouse NFAT-c1: 5′-CCAGTATACCAGCTCTGCCA-3′, 5′-GTGGGAAGTCAGAAGTGGGT-3′; mouse DC-STAMP: 5′-TACGTGGAGAGAAGCAAGGAA-3′, 5′-ACACTGAGACGTGGTTTAGGAAT-3′; mouse β-actin: 5′-TCTGGCACCACACCTTCTAC-3′, 5′-TACGACCAGAGGCATACAGG-3′; human CTSK: 5′- GAGGCTTCTCTTGGTGTCCATAC-3′, 5′-TTACTGCGGGAATGAGACAGGG-3′; human M-CSF: 5′-GAAAGTTTGCCTGGGTCCTC-3′, 5′-GATGGCATTGGGGGTGTTAT-3′; human RANKL: 5′-ATGCGGTTTGCAGTTCTTCTC-3′, 5′-ACTCCTTATCTCCACTTAGG-3′; human OPG: 5′-ACGGCAACACAGCTCACAAG-3′, 5′-AAGCTACGAAGCTGCTCGAAG-3′; human β-actin -: 5′-ACTCTTCCAQGCCTTCCTTCC-3′, 5′-TGTTGGCGTACAGGTCTTTG-3′.

### 4.7. Transwell Co-Culture Osteoclastogenesis System

The co-culture systems were established with 24-well plates containing transwell inserts (0.4-μm pore size). The BMMs were seeded in the lower chamber at a density of 2 × 10^4^ cells/well. To induce osteoclastogenesis, BMMs were treated with 100 ng/mL of RANKL every other day for a 4-day period. The insert was located in a neighboring well coated with collagen TYPE I, where PDLFs were seeded at a density of 2 × 10^4^ cells/well. In case of siRNA transfection, it was conducted when the PDLF were seeded. PDLFs were treated with 10 ng/mL of TNFα for 24 h and washed with PBS to remove the excess TNFα prior to transferring the insert to BMM-seeded wells. After 48 h RANKL treatment on BMMs, PDLF-seeded upper chamber was transferred onto BMM-seeded lower chamber

### 4.8. Transfection with siRNA

The siRNAs for human CTSK and non-specific control siRNA were purchased from GenePharma (Shanghai, China): siCTSK 5′- CCAUAACCUUGAGGCUUCUTT-3′ (sense), 5′- AGAAGCCUCAAGGUUAUGGTT-3′ (antisense); siControl 5′- UUCUCCGAACGUGUCACGUTT-3′ (sense), 5′- ACGUGACACGUUCGGAGAATT-3′ (antisense). The jetPRIME reagent (Polyplus, New York, USA) was used to transfect siRNA. Briefly, the respective siRNAs were diluted in jetPRIME buffer and then jetPRIME reagent was added. After incubation for 10 min, the transfection mix was added dropwise to the cells. After incubation of PDLFs with transfection reagent containing siRNA for 6 h, the transfection medium was changed with fresh medium, and the expression of genes of interest was determined using qPCR.

### 4.9. Resorption Assay

To assess the bone-resorbing activity of osteoclasts, BMMs were cultured on a commercially available 24-well plate bone resorption assay kit (#CSR-BRA-24KIT, Cosmo Bio, Tokyo, Japan) according to the manufacturer’s instructions. BMMs were suspended in phenol red-free α-MEM containing 10% FBS and plated at a density of 2 × 10^4^ cells/well. BMMs were treated with 100 ng/mL of RANKL to induce osteoclastogenesis, and the medium was replaced every other day. On day 2, PDLF-seeded transwell inserts were transferred onto BMM-seeded lower chamber. On day 6, the fluorescence intensity was measured at an excitation wavelength of 485 nm and an emission wavelength of 535 nm. To measure the pit areas, the cells were removed with 5% sodium hypochlorite and photographed using a light microscope.

### 4.10. Statistical Analysis

All data were obtained from at least three independent experiments conducted in triplicate. Results of multiple observations are presented as mean ± S.E.M. Comparisons between two groups were performed using Student’s *t*-test. For analyzing multivariate data, group differences were assessed using one-way or two-way ANOVA, followed by Bonferroni post-hoc test.

## Data Availability

The data presented in this study are available within this article.

## Abbreviations

CTSK	Cathepsin K
PD	Periodontal disease
TNFα	Tumor necrosis factor-alpha
PDLF	Periodontal ligament fibroblast
DPSC	Dental pulp stem cell
GF	Gingival fibroblast
RANKL	Receptor activator of nuclear factor kappa-B ligand
M-CSF	Macrophage-colony stimulating factor
OPG	Osteoprotegerin
BMM	Bone marrow-derived macrophage
TRAP	Tartrate-resistant acid phosphatase
